# Supplementary material for: OraSure InteliSwab™ Rapid Antigen Test Performance with the SARS-CoV-2 Variants of Concern—Alpha, Beta, Gamma, Delta, and Omicron
Source: Viruses. 2022 Mar 6;14(3):543. doi: 10.3390/v14030543 (PMC8951130; doi:10.3390/v14030543)
Supplement: Supplementary file 1 [file viruses-14-00543-s001.zip › viruses-1610721-supplementary.pdf]

viruses

MDPI

Supplementary Materials

OraSure InteliSwab™ Rapid Antigen Test Performance with the SARS-CoV-2 Variants of Concern—Alpha, Beta, Gamma, Delta, and Omicron

Zachary A. Weishampel [1], Janean Young [2], Mark Fischl [2], Robert J. Fischer [1], Irene Owusu Donkor [1,3], Jade C. Riopelle [1], Jonathan E. Schulz [1], Julia R. Port [1], Taylor A. Saturday [1], Neeltje van Doremalen [1], Jody D. Berry [2], Vincent J. Munster [1,*] and Claude Kwe Yinda [1]

Table S1. SARS-CoV-2 isolates used in this study.

Virus	WHO Description	PANGO Lineage	GiSAID/Genbank Acc
SARS-CoV-2/human/USA/WA-CDC-WA1/2020	Lineage A	WA1	MN985325
England/204820464/2020	Alpha	B.1.1.7	EPI_ISL_683466
USA/MD-HP01542/2021	Beta	B.1.351	EPI_ISL_890360
hCoV-19/USA/GA-EHC-2811C/2021	Gamma	P1	GISAID EPI_ISL_7171744
hCoV-19/USA/KY-CDC-2-4242084/2021	Delta	B.1.617.2	EPI_ISL_1823618
hCoV-19/USA/WI-WSLH-221686/2021	Omicron	B.1.1529	EPI_ISL_7263803

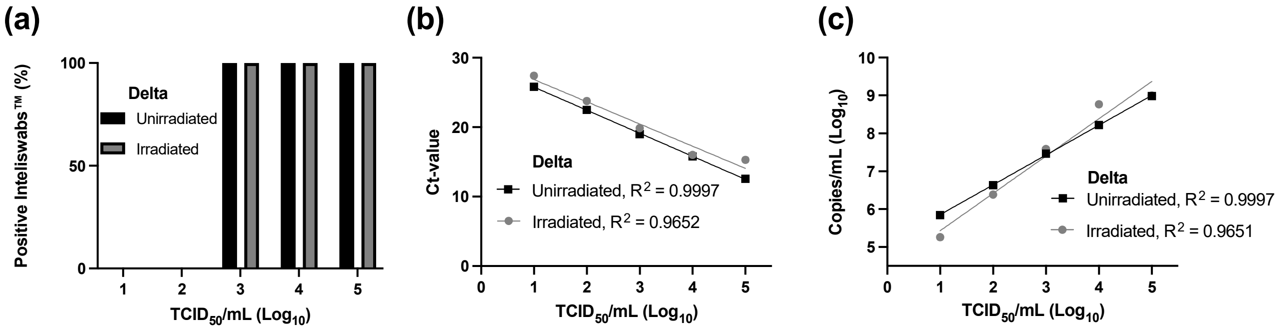

Figure S1. Evaluation of the effects of irradiation on OraSure InteliSwab™ results. Two 10-fold dilutions, beginning at 1.00×10^5 $TCID_{50}$/mL, were performed with live Delta and irradiated Delta. For each dilution, 50 μL was applied to 3 separate OraSure InteliSwab™. Irradiation dosage was 2 Mrad. **(a)** Bar plots showing percentage positive OraSure InteliSwab™ results for live versus irradiated Delta variant 10-fold dilutions. **(b)** Scatter plot depicting Ct-values for live versus irradiated Delta variant 10-fold dilutions. **(c)** Scatter plot showing RNA copy concentration for live versus irradiated Delta variant 10-fold dilutions.

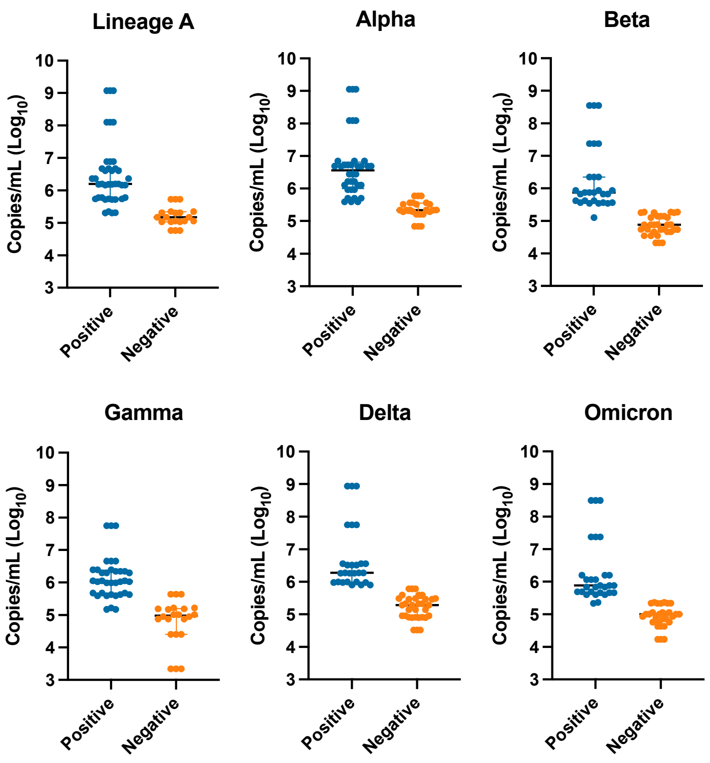

Figure S2. Distribution of all OraSure InteliSwab™ results tested on irradiated sample compared to the RNA copies/mL. Scatter plots represent all OraSure InteliSwab™ results on irradiated 10-fold and 2-fold dilutions for each variant tested, including lineage A, Alpha, Beta, Gamma, Delta, and Omicron. The blue and orange lines represent the interquartile range for each positive or negative group. The black line represents median.
